# High-resolution tandem mass spectrometry dataset reveals fragmentation patterns of cardiac glycosides in leaves of the foxglove plants

**DOI:** 10.1016/j.dib.2020.105464

**Published:** 2020-03-20

**Authors:** Baradwaj Gopal Ravi, Mary Grace E. Guardian, Rebecca Dickman, Zhen Q. Wang

**Affiliations:** a Department of Biological Sciences, University at Buffalo, State University of New York, Buffalo, NY 14260, United States; b Department of Chemistry, Chemistry Instrumentation Center, University at Buffalo, State University of New York, Buffalo, NY 14260, United States

**Keywords:** Cardenolides, *Digitalis purpurea*, *Digitalis lanata*, Digoxin, Digitoxin, Heart failure

## Abstract

Cardiac glycosides, steroid derivatives extracted from the foxglove plants, have been used for the treatment of heart failure since the 18th century. A method based on liquid chromatography coupled with high-resolution tandem mass spectrometry (LC/MS^2^) has been developed to characterize and quantify cardiac glycosides in fresh-leaf extracts of the foxglove (*Digitalis sp.*) plants [1]. In this report, the fragmentation spectra of additional authentic standards of cardiac glycoside (digitoxigenin, digoxigenin, *β*-acetyldigoxin) and cardenolides identified in the leaves of *Digitalis lanata* (*D. lanata*) and *Digitalis purpurea* (*D. purpurea*) were provided with high resolution. The exact mass of signature peaks for the aglycones and the sugar units of cardenolides were measured. This dataset is valuable to researchers interested in characterizing cardenolides in plants, or quantifying cardenolides in drug tablets, or studying cardenolide toxicities in animals. The fragmentation patterns of authentic cardenolide standards provided in these data can be used to validate relevant cardenolides in various biological samples and to infer chemical structures of unknown cardiac glycosides.

**Specifications table**

**Table:** 

Subject	Plant science
Specific subject area	Biochemistry, Metabolomics
Type of data	Table
	Figure
How data were acquired	Thermo Scientific Q-Exactive Focus^TM^ liquid-chromatography tandem mass spectrometry system coupled with a high-resolution Orbitrap^TM^ analyser.
Data format	Raw
	Analyzed
Parameters for data collection	The first pair of true leaves were collected from 5-week-old *D. lanata* and *D. purpurea* grown at 28°C in a growth chamber. The leaves were frozen immediately in liquid nitrogen and kept at -80°C for further analysis.
Description of data collection	Freeze-dried leaf samples were extracted with 80% methanol before injecting into the LC/MS^2^ system. The extract was separated on a C18 column before the MS analysis by the Q-Exactive^TM^. A selected ion monitoring with a data-dependent MS^2^ (SIM-ddMS^2^) was used for data acquisition.
Data source location	Chemistry Instrumentation Center,
	University at Buffalo, State University of New York,
	Buffalo, New York,
	United States
Data accessibility	Analyzed data: in the article
	Raw Data:
	Repository name: Mendeley Data
	Data identification number: 10.17632/y7p9tbh2nt.1 Direct URL to data: https://data.mendeley.com/datasets/y7p9tbh2nt/1
Related research article	Ravi, B.G., Guardian, M.G.E., Dickman, R., Wang, Z.Q., Profiling and structural analysis of cardenolides in two species of *Digitalis* using liquid chromatography coupled with high-resolution mass spectrometry, Journal of Chromatography A, January 2020, 460903, In Press, https://doi.org/10.1016/j.chroma.2020.460903

## Value of the data

• Providing fragmentation patterns of various cardiac glycosides that are currently absent in major spectral libraries such as the Global Natural Product Social Molecular Networking and Metlin [2,3].
• Academic, pharmaceutical, and clinical researchers interested in characterizing cardenolides in plants, or quantifying cardenolides in digoxin drug tablets, or studying cardenolide toxicity in animals will benefit from these data.
• The fragmentation patterns of authentic cardenolide standards provided in these data can be used to validate relevant cardenolides in various biological samples and to infer chemical structures of unknown cardiac glycosides.
• The fragmentation patterns of cardenolides extracted from *D. lanata* and *D. purpurea* can be used to further examine the complete profiles of cardiac glycosides in different species of plants.

## 1 Data description

### 1.1 Fragmentation patterns of cardiac glycoside authentic standards

**Fig. 1:**
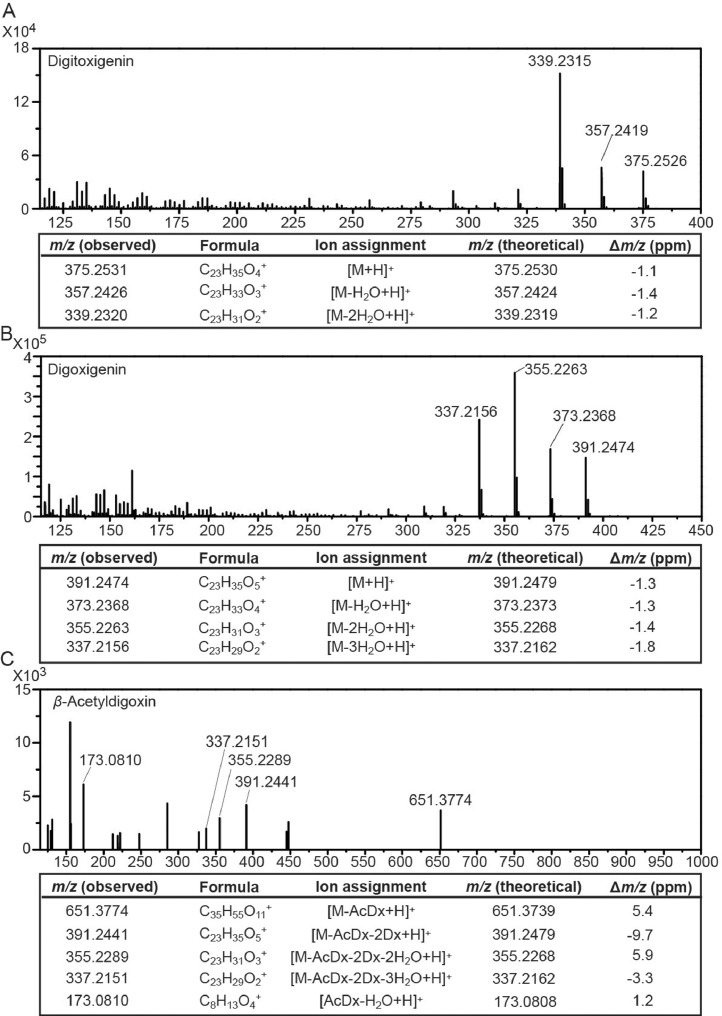
MS^2^ product ion mass spectra from [M+H]^+^ adducts of digitoxigenin (A), digoxigenin (B), and *β*-acetyldigoxin (C) standards with putative ion structures. I.C.: ion counts; ppm: parts per million; Dx: digitoxose unit.

To investigate how cardenolides behave during collision-induced dissociation (CID) in tandem mass (MS^2^) spectrometry, available authentic standards were analyzed. A 17-min method using liquid chromatography (LC) coupled with a high-resolution Orbitrap^TM^ analyzer was developed. In the full-scan mode, both the [M+H]^+^ and the [M+Na]^+^ parent ions were detected for each pure compound within five ppm units of the theoretical exact mass. The [M+H]^+^ adducts fragmented well during collision, and comprehensive MS^2^ spectra were generated (Fig. 1). Three characteristic peaks, including *m/z* 375.2531, *m/z* 357.2426, and *m/z* 339.2320 were detected in the MS^2^ spectrum of the aglycone digitoxigenin, all within five ppm units of the theoretical exact mass (Fig 1A). These peaks featured sequential dehydration of the two hydroxyl groups in digitoxigenin. The same peaks were observed in digitoxin pure standard, a cardenolide with digitoxigenin aglycone and three digitoxose moieties covalently linked to the 3-hydroxyl group of the aglycone [1]. The MS^2^ spectrum of the aglycone digoxigenin showed four signature peaks: *m/z* 391.2474, *m/z* 373.2368, *m/z* 355.2263, and *m/z* 337.2156, resulting from the sequential dehydration of three hydroxyl groups (Fig. 1B). The same four peaks were also observed in the MS^2^ spectra of cardenolides with the same aglycone, such as *β*-acetyldigoxin and lanatoside C (Fig. 1C) [1]. Two additional peaks were shown in the fragmentation profile of *β*-acetyldigoxin. The *m/z* 651.3774 peak corresponded to the *β*-acetyldigoxin without the terminal *β*-acetyldigitoxose moiety, and the *m/z* 173.0810 peak corresponded to the dehydrated product of the *β*-acetyldigitoxose. The raw data of the chromatograms and the MS^2^ spectra of cardiac glycoside standards can be downloaded from the Mendeley Database.

### 1.2 *Fragmentation patterns of cardiac glycosides identified in the leaves of* D. lanata *and* D. purpurea

**Fig. 2:**
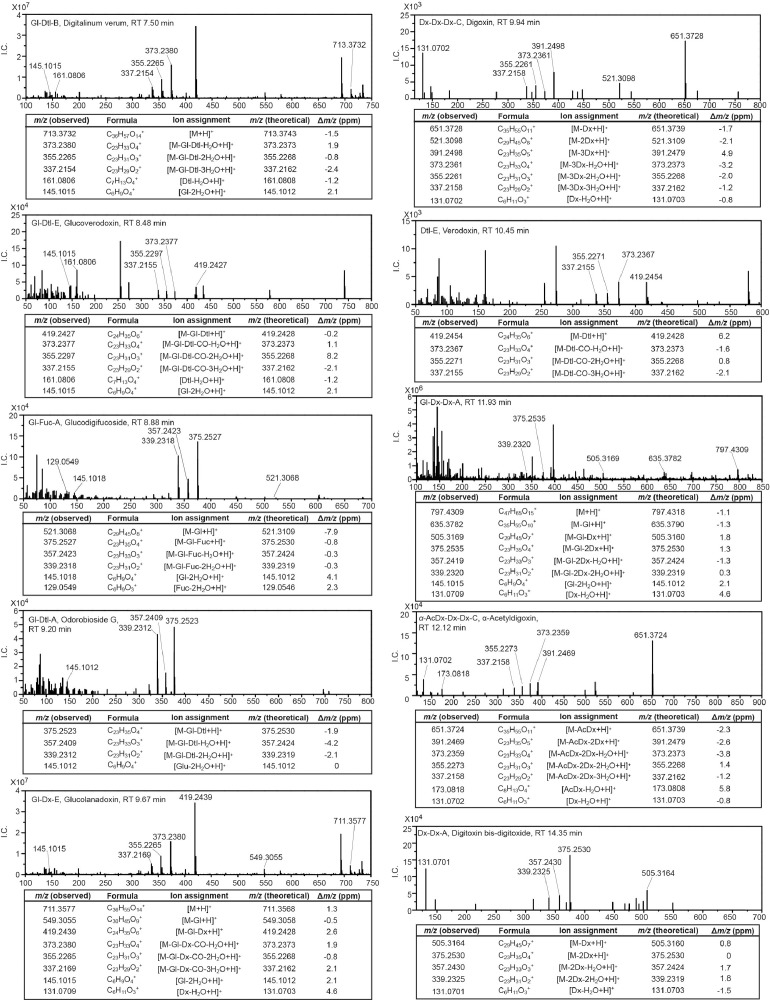

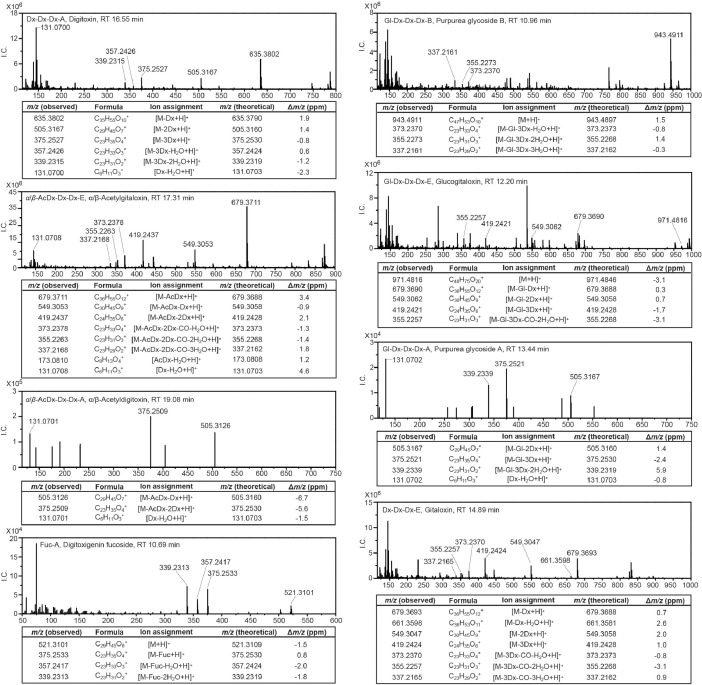
MS^2^ product ion mass spectra from [M+H]^+^ adducts of cardenolides identified in *D. lanata* and *D. purpurea*. I.C.: ion counts; ppm: parts per million; RT: retention time; Aglycones: A, digitoxigenin; B, gitoxigenin; C, digoxigenin; D, diginatigenin; E, gitaloxigenin. Sugars: AcDx, acetyldigitoxose; Dx, digitoxose; Gl, glucose; Dtl, digitalose; Fuc, fucose.

*D. lanata* and *D. purpurea* have been the most frequently studied species in *Digitalis* genus that produce cardiac glycosides [4]. To analyze cardiac glycosides in the leaves of *D. lanata* and *D. purpurea*, a 60-min method of liquid chromatography coupled with high-resolution tandem mass spectrometry was developed for better separation of compounds in crude plant extracts. Both the full scan and the selected ion monitoring with data-dependent tandem mass spectrometry (full MS-ddMS^2^ and SIM-ddMS^2^) methods were utilized to ensure the fragmentation of less-abundant cardenolides in leaf samples. As with the pure standards, both the [M+H]^+^ and the [M+Na]^+^ adducts were detected with a Δ*m/z* less than ten ppm units for all identified cardiac glycosides. The [M+H]^+^ parent ions were easily fragmented during the CID, and the fragmentation patterns revealed the signature peaks for both the aglycones and the sugar units. These signature peaks formed the basis for annotating cardenolides in *Digitalis* (Fig. 2).

The theoretical mass of signature peaks for each class of aglycones in *Digitalis* was distinct: digitoxigenin (abbreviated as A): *m/z* 375.2530, *m/z* 357.2424, *m/z* 339.2319; gitoxigenin (B): *m/z* 373.2373, *m/z* 355.2268, *m/z* 337.2162; digoxigenin (C): *m/z* 391.2497, *m/z* 373.2373, *m/z* 355.2268, *m/z* 337.2162; gitaloxigenin (E): *m/z* 419.2428, *m/z* 373.2373, *m/z* 355.2268, *m/z* 337.2162. Peaks observed from crude leaf extracts of *Digitalis* were within ten ppm units of the theoretical exact mass.

The characterization of sugar units in cardenolides was more challenging due to their smaller sizes. Nevertheless, the theoretical mass of signature peaks for the sugar moieties present in cardenolides were: digitoxose (Dx): *m/z* 131.0703; acetyldigitoxose (AcDx): *m/z* 173.0808; digitalose (Dtl): *m/z* 161.0808; glucose (Gl): *m/z* 145.1012; fucose (Fuc): *m/z* 129.0546. As with the aglycones, the *m/z* of the observed peaks of *Digitalis* extract were within ten ppm units of the theoretical exact mass. For some cardenolides, such as odorobioside G (Gl-Dtl-A) and digitoxigenin fucoside (Fuc-A), the sugar moieties were inferred from the neutral loss of mass between fragments. The signature peaks for the sugars identified corresponded well with the predicted spectra using the CFM-ID software [5,6].

Overall, seventeen and seven cardenolides were identified unambiguously in *D. lanata* and *D. purpurea*, respectively [1]. It is worthwhile to mention that structural isomers are not always distinguishable even with a high-resolution LC/MS^2^ system due to the same exact mass and fragmentation patterns. Therefore, with no authentic standards, structural isomers including *α/β-*acetylgitaloxin and *α/β-*acetyldigitoxin were not distinguished in this dataset. The *α*- isomer of acetyldigoxin was assigned since its retention time was different from the available *β*-acetyldigoxin authentic standard. The raw data of the chromatograms and the MS^2^ spectra of cardiac glycoside identified in leaves of *Digitalis* can be downloaded from the Mendeley Database.

## 2 Experimental design, materials, and methods

### 2.1 Chemicals

Digitoxigenin, digoxigenin, *β*-acetyldigoxin were purchased from the Millipore Sigma (St. Louis, Missouri, United States). 0.1% formic acid in water and 0.1% formic acid in acetonitrile were acquired from Honeywell International Inc (Muskegon, Michigan, United States).

### 2.2 Plant materials

*D. lanata* and *D. purpurea* seeds were procured from Strictly Medicinal (Williams, Oregon, USA) and stored in dry and dark conditions at room temperature. Seeds were sowed in the garden soil mix, and germination was observed after ten days. The seedlings were kept in a growth chamber (Invitrogen, Clayton, Missouri, USA) at 25˚C under a 16-h light, 8-h dark period with a relative humidity of 60–80%. The pair of the first true leaves were harvested five weeks after germination and were frozen immediately in liquid nitrogen followed by storing in -80˚C before extraction.

### 2.3 Preparation of plant extract

The frozen leaves of *D. lanata* and *D. purpurea* were lyophilized using a Labconco FreeZone 2.5 lyophilizer (Kansas, Missouri, USA) for 24 h. Further, the samples were individually homogenized in 1.5 ml tubes using polypropylene pellet pestles (DWK life sciences, NJ, USA). The homogenized samples were resuspended in 80% methanol at a final concentration of 10 mg/ml. The resuspended samples were vortexed briefly and incubated for 10 min at 65˚C. Then, the extract was centrifuged at 18,000 g for 10 min to sediment the leaf debris. The supernatant was filtered through a 96-well 0.45 µm MultiScreenHTS filter plate (Merck Millipore, Carrigtwohill, Ireland). Finally, the samples were frozen at -20˚C pending LC/MS analysis.

### 2.4 LC-HRMS analysis for cardenolide standards

Liquid chromatography coupled with tandem high-resolution mass spectrometry (LC - HRMS) analysis was carried out using a Thermo Scientific Q-Exactive Focus^TM^ with Thermo Scientific UltiMate 3000 UHPLC^TM^. A Waters XSelect CSH^TM^ C18 HPLC column with a particle size of 3.5 µm, an internal diameter of 2.1 mm and a length of 150 mm was used for the separation. Mobile phases were water with 0.1% formic acid (mobile phase A) and acetonitrile with 0.1% formic acid (mobile phase B) operated at a flow rate of 200 µL min^-1^. The table below summarizes the linear gradient program used in the analysis.

**Table:** 

Time (min)	%Mobile phase A	%Mobile phase B
0	90	10
2	90	10
12	5	95
14	5	95
15	90	10
17	90	10

The sample injection volume was 10 µL. The analysis was done in the positive mode (+ESI) using a full-scan with a data-dependent MS^2^ (full MS-ddMS^2^) method with an inclusion list containing the parent ion mass of targeted analytes. The following parameters were used to collect the HRMS data:

**Table:** 

Parameters	Settings
Scan range	*m/z* 100 - 1200
Resolution	70, 000 (full MS); 17,500 (ddMS^2^)
Isolation window	*m/z* 4.0
AGC target	1E^6^
Injection time	auto
Collision energy	10V, 30V, 60 V

### 2.5 LC-HRMS analysis of cardenolides in digitalis leaf extract

To investigate the presence of cardenolides in the leaf extract, the chromatographic method described above was extended for a better separation. The gradient initiated from 10% mobile phase B, followed by a 5-min linear increase to 30%, and further increased to 70% at 20 min followed by holding for 10 min. This was further followed by a 10-min linear gradient to 95% mobile phase B, held for 10 min and was returned to the initial conditions within 2 min. An 8-min equilibration was employed prior to the next injection. The inclusion list was modified to include all suspected cardenolides that could be in the sample. For less abundant cardenolides identified from the full MS-ddMS^2^ method with precursor ion and isotope match, MS^2^ spectra were obtained using a selected ion monitoring with MS^2^ (SIM-ddMS^2^). In this case, the inclusion list specified the retention time of all of the suspected cardenolides obtained in the full MS-ddMS^2^. Similar HRMS settings were used as above except that the resolution for SIM was set to 35,000, and the AGC target was set to 5E^4^. Data processing involved extracting the precursor ions within five ppm units of the theoretical mass of the suspected cardenolides with MS^2^ spectra and retention time match.

## Declaration of Competing Interest

The authors declare that they have no known competing financial interests or personal relationships which have, or could be perceived to have, influenced the work reported in this article.
